# Treatment Outcome of 227 Patients with Sinonasal Adenoid Cystic Carcinoma (ACC) after Intensity Modulated Radiotherapy and Active Raster-Scanning Carbon Ion Boost: A 10-Year Single-Center Experience

**DOI:** 10.3390/cancers11111705

**Published:** 2019-11-01

**Authors:** Sati Akbaba, Dina Ahmed, Andreas Mock, Thomas Held, Suzan Bahadir, Kristin Lang, Mustafa Syed, Juliane Hoerner-Rieber, Tobias Forster, Philippe Federspil, Klaus Herfarth, Peter Plinkert, Juergen Debus, Sebastian Adeberg

**Affiliations:** 1 Department of Radiation Oncology, University Hospital Heidelberg, Im Neuenheimer Feld 400, 69120 Heidelberg, Germany; dina.ahmed@med.uni-heidelberg.de (D.A.); thomas.held@med.uni-heidelberg.de (T.H.); kristin.lang@med.uni-heidelberg.de (K.L.); mustafa.syed@med.uni-heidelberg.de (M.S.); juliane.hoerner-rieber@med.uni-heidelberg.de (J.H.-R.); tobias.forster@med.uni-heidelberg.de (T.F.); klaus.herfarth@med.uni-heidelberg.de (K.H.); juergen.debus@med.uni-heidelberg.de (J.D.); sebastian.adeberg@med.uni-heidelberg.de (S.A.); 2 Heidelberg Institute of Radiation Oncology (HIRO), Im Neuenheimer Feld 400, 69120 Heidelberg, Germany; 3 National Center for Tumor Diseases (NCT), 69120 Heidelberg, Germany; 4 Heidelberg Ion-Beam Therapy Center (HIT), Im Neuenheimer Feld 450, 69120, 69120 Heidelberg, Germany; 5 Department of Medical Oncology, National Center for Tumor Diseases (NCT) Heidelberg, Heidelberg University Hospital, Im Neuenheimer Feld 460, 69120 Heidelberg, Germany; andreas.mock@med.uni-heidelberg.de; 6 Department of Radiology, University Hospital Heidelberg, Im Neuenheimer Feld 400, 69120 Heidelberg, Germany; dr_suzanb@hotmail.com; 7 Department of Radiology, Koru Hospitals-Yuksek Ihtisas University, 06520 Ankara, Turkey; 8 Clinical Cooperation Unit Radiation Oncology, German Cancer Research Center (DKFZ), Heidelberg, Germany; 9 Department of Otorhinolaryngology, University Hospital Heidelberg, Im Neuenheimer Feld 400, 69120 Heidelberg, Germany; philippe.federspil@med.uni-heidelberg.de (P.F.); peter.pinkert@med.uni-heidelberg.de (P.P.)

**Keywords:** carbon ion radiotherapy, adenoid cystic carcinoma, sinonasal carcinoma, local control, recurrence patterns, toxicity

## Abstract

We aimed to evaluate the treatment outcome of primary and postoperative bimodal radiotherapy (RT) including intensity modulated photon radiotherapy (IMRT) and carbon ion radiotherapy (CIRT) for sinonasal adenoid cystic carcinoma (ACC) patients. Medical records of 227 consecutive patients who received either a primary (*n* = 90, 40%) or postoperative (*n* = 137, 60%; R2, *n* = 86, 63%) IMRT with doses between 48 and 56 Gy in 1.8 or 2 Gy fractions and active raster-scanning carbon ion boost with 18 to 24 Gy (RBE, relative biological effectiveness) in 3 Gy (RBE) fractions between 2009 and 2019 up to a median total dose of 80 Gy (EQD2, equivalent dose in 2 Gy single dose fractions, range 71–80 Gy) were reviewed. *Results*: Median follow-up was 50 months. In univariate and multivariate analysis, no significant difference in local control (LC) could be shown between the two treatment groups (*p = 0.33*). Corresponding 3-year LC rates were 79% for primary bimodal RT and 82% for postoperative bimodal RT, respectively. T4 stage (*p = 0.002*) and solid histology (*p = 0.005*) were identified as independent prognostic factors for decreased LC. Significant worse long-term treatment tolerance was observed for postoperatively irradiated patients with 17% vs. 6% late grade 3 toxicity (*p < 0.001*). Primary radiotherapy including IMRT and carbon ion boost for dose-escalation results in adequate LC with less long-term grade 3 toxicity compared to postoperative bimodal radiotherapy in sinonasal ACC patients. The high rate of macroscopic tumor disease in the postoperative group makes the interpretation of the beneficial results in LC for primary RT difficult.

## 1. Introduction

Adenoid cystic carcinomas (ACCs) account for 5% of all malignant sinonasal tumors and correspond to less than 0.15% of all malignancies in the head and neck [1,2,3]. With an incidence of 25% ACCs belong to one of the most common histological subtypes of malignant salivary gland tumors (MSGTs) which are divided into MSGTs of the minor or major salivary glands [4]. ACCs are characterized by a slow but locally aggressive growth pattern along involved cranial nerves (perineural invasion, PNI) and a high rate of hematogenous metastases, even years after first diagnosis [4,5,6].

Standard treatment for these tumors consists of complete resection and postoperative radiotherapy (RT) in case of risk factors, i.e. incomplete resection margins (R1/2), PNI, advanced tumor stages (T3/4), although several authors could show, that all tumor stages (T1-4) profit from postoperative RT [5,7,8,9,10]. Nevertheless, ACCs are preferentially diagnosed in inoperable stages due to the late occurrence of symptoms, so that an extensive resection with a high morbidity rate or primary RT is required in most cases [11,12]. While high RT doses >80 Gy are needed for tumor control due to the relative radio resistance of these tumors, dose escalation especially in the area of the paranasal sinuses and the nasal cavity is often limited in the head and neck region by surrounding organs at risk [5,13,14]. Several authors describe better LC rates for postoperative RT compared to RT alone in ACC patients [8,15]. Nevertheless, due to patient selection bias, i.e. T stage, N stage, operable vs. inoperable tumors, patient condition, age, the benefit of postoperative RT compared to definitive RT still remains unclear.

While LC rates between 38% and 64% are shown for photon beam RT, high-LET radiotherapy can achieve superior LC due to a higher relative biological effectiveness [4,5,13,16,17,18,19,20]. As it is known, ACCs are relatively radio resistant tumors requiring high-doses for local tumor control [5,14]. Thus, a 5-year LC of 50% to 70% could be achieved with protons and even 93% with neutrons [16,17,18,19,20]. In a randomized phase-III trial, Laramore et al. reported a significantly higher 10-year locoregional control of 56% vs. 17% for neutrons vs. photons [16]. First experiences with CIRT (total dose between 52.8 and 70.2 Gy (RBE) in 16 or 18 fractions over 4–6 weeks) for head and neck ACC (overall 9 patients) by the NIRS in Chiba showed a 5-year LC rate of 50% for these tumors [21]. Further experiences were reported by the Society of Heavy Ion Research (GSI, Darmstadt, Germany) in cooperation with the Department of Radiation Oncology, University Hospital Heidelberg regarding CIRT boost (18 Gy (RBE)/3 Gy (RBE)) in combination with IMRT (54 Gy/1.8 Gy) within a phase -I/II study [22]. In this trail, Schulz-Ertner et al. showed superior 4-year LC control rates of 77.5% vs. 24.6% for bimodal RT vs. photon beam RT regarding high-risk ACC of the head and neck [22,23]. In the prospective COSMIC-trial as well, Jensen et al. could show favorable treatment outcome for bimodal RT (CIRT with 24 Gy (RBE)/3 Gy (RBE) and IMRT with 50 Gy/2 Gy) with a 3-year LC, PFS and OS of 81.9%, 57.9% and 78.4% for MSGTs (89% ACC and 34% tumors of the paranasal sinuses), comparable with Japanese data for CIRT alone [24,25,26,27,28]. According OS, a 5-year OS between 55% and 85% is described for all RT modalities in the literature [14,18,19,25,29].

In the current study, we compared treatment outcome of 227 patients with sinonasal ACC, who received either primary or postoperative bimodal RT. The current patient collective represents, to our best knowledge, the largest patient collective reported so far for paranasal ACC.

## 2. Methods and Materials

### 2.1. Evaluation

Medical records of patients with ACC of the paranasal sinuses and the nasal cavity, who were treated between 2009 and 2019 with bimodal radiotherapy including intensity modulated photon radiotherapy (IMRT) and carbon ion radiotherapy (CIRT) and received regular follow-up examinations in our institution, were analyzed retrospectively. Overall, 227 patient data were available for the statistical analysis.

Overall survival (OS) and distant progression-free survival (DPFS) were calculated from the first diagnosis to the last follow-up or time of event (death/distant progression). LC was assessed from time of RT completion up to time of local progression. Kaplan-Meier estimates of potential prognostic factors were compared using the log-rank test for univariate analysis and the cox-regression model for multivariate analysis. Patient and treatment characteristics for primary vs. postoperative RT were compared with the Fisher’s Exact Test for categorial data and the Wilcoxon signed-rank test for continuous data. A *p*-value of < 0.05 was considered as statistically significant. Statistical tests were conducted with SPSS Statistics version 24 (IBM, Armonk, NY, USA) and R version 3.4.2. (www.r-project.org). 

Toxicity according to the Common Terminology Criteria for Adverse Events (CTCAE) and tumor response according to the Response Evaluation Criteria in Solid Tumors (RECIST) on the basis of regularly performed magnetic resonance imaging (MRI) were assessed at six different time points (during RT+6 weeks post RT for acute toxicity and three, six, 12 and 24 months post-RT as well as at last follow-up for late toxicity and RECIST analysis).

### 2.2. Patient Characteristics

Overall, 39.6% of the patients received primary (*n* = 90) and 60.4% postoperative bimodal RT (*n* = 137). Patient and tumor characteristics for the whole cohort, for primary bimodal and postoperative bimodal RT are presented in Table 1. Additionally, characteristics between the two treatment groups of primary and postoperative bimodal RT were statistically compared (for *p-*values please see Table 1). 

### 2.3. Treatment Planning and Treatment Characteristics

Before RT start, a CT scan in head-first supine position was performed (immobilization with thermoplastic head masks). Target delineation was based on a current MRI and in case of postoperative RT a preoperative MRI was additionally used for tumor demarcation. Routinely, a T1- and T2-weighted MRI sequence with and without contrast substance including a T2 STIR (short tau Inversion recovery) and a T2 FLAIR (fluid-attenuated Inversion recovery) sequence was matched to the CT scan in irradiation position in form of a rigid co-registration. Treatment planning was performed with Syngo PT Planning, Version 13 (Siemens, Erlangen, Germany) for CIRT and TomoTherapy®-Planning Station (Accuray, Sunnyvale, CA, USA), a special form of photon IMRT with helical treatment fields and an on-board imaging capability (MVCT), for photon radiotherapy [30]. Two different clinical target volumes were outlined; CTV2 included the macroscopic tumor or tumor bed and CTV1 included CTV2 and typical local and regional pathways (retropharyngeal lymph nodes + cervical lymph node levels I–III) of tumor spread. The unilateral cervical lymphatic drainage was involved into the CTV1 (93.2%) in case of N+ and electively in case of T3/T4 tumors. In case of midline infiltrating tumors the bilateral cervical lymphatic drainage was included into the CTV1. Planning target volumes (PTVs) were defined by adding an isotropic margin of 3 mm to the CTVs. PTV margin was reduced in the area of tumor extension into critical structures. Optic chiasm, optical nerves, brain stem and spinal cord were defined as critical structures without a safety margin. Photon RT was performed with five fractions per week to the PTV1 with 48 Gy to 56 Gy in 1.8 Gy or 2.0 Gy fractions. Carbon ion boost to the PTV2 was applied in intensity-controlled active raster-scanning technique with 5-6 fractions in 3 Gy (RBE, relative biological effectiveness) fractions per week up to a median total EQD2 of 80 Gy (range 71–80 Gy). Doses for primary and postoperative RT were similar as the majority of postoperatively irradiated patients were treated for macroscopic residual disease (R2, 63%). Photon as well as carbon ion doses were prescribed to the median PTV and covered by the 95% prescription isodose. Critical structures were spared according to the QUANTEC data as low as possible [31,32]. At sites where the tumor extended into the critical structures, doses were reduced without affecting PTV coverage. For detailed treatment characteristics, please see Table 1.

### 2.4. Declarations

(1) Ethics approval and consent to participate: The final protocol was approved by the ethics committee of the University of Heidelberg, Germany (S-421/2015). (2) Consent for publication: Not applicable. (3) Availability of data and material: All data generated or analysed during the current study are included in this published article. The dataset is available from the corresponding author on reasonable request. (4) Statistical analyses: Sati Akbaba and Andreas Mock are responsible for statistical analysis.

## 3. Results

**Figure 1:**
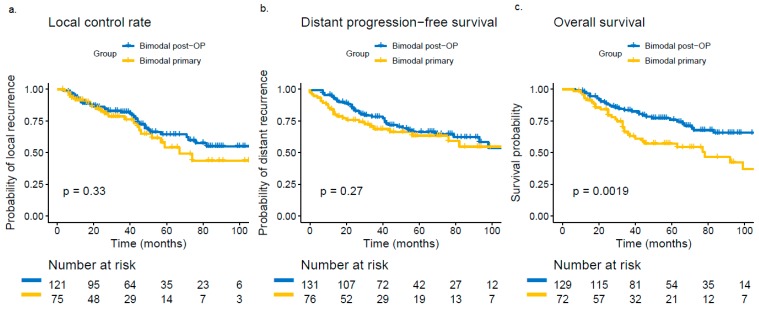
Kaplan-Meier estimates of local control (**a**., *p* = 0.33), distant progression-free survival (**b**., *p* = 0.27) and overall survival (**c**., *p* < 0.01) for primary and postoperative bimodal radiotherapy.

**Figure 2:**
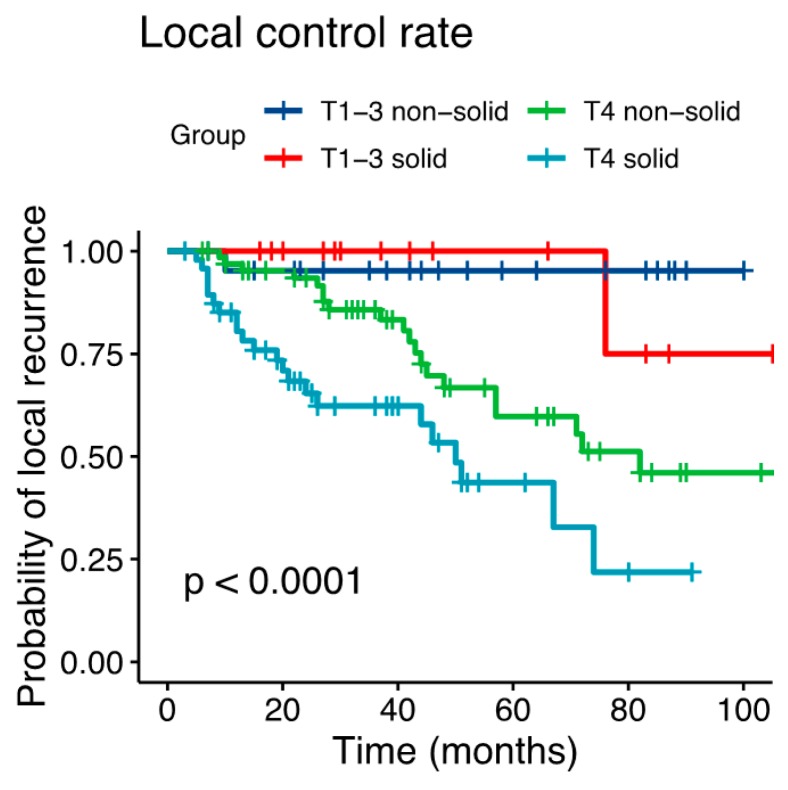
Kaplan-Meier estimates of local control depending on T stage and solid vs. non-solid histology (*p* < 0.0001).

Median follow-up was 50 months (range 3–109 months). At last follow-up, 172 patient data (75.8%) were available for RECIST analysis as 32 patients (14.1%) were lost to follow-up and 23 patients (10.1%) were dead without local recurrence (for detailed RECIST analysis, please see Table S1). Overall, 61 of these patients showed local relapse at last follow-up (35.5%). In the univariate and multivariate analysis, we could not reveal any statistically significant differences in the LC rate for the primary vs. the postoperative bimodal RT group (*p* = 0.859; Table 2 and Table S2). Corresponding Kaplan-Meier estimates showed a 3-year LC of 79% for primary vs. 82% for postoperative RT and an estimated 5-year LC of 58% for the primary vs. 65% for the postoperative RT group (*p* = 0.33, Figure 1). T4 stage (vs. T3/2/1; *p = 0.002*) and solid histology (vs. non-solid histology; *p* = 0.005) could be identified as independent negative prognostic factors for LC in the multivariate model (Table 2). Kaplan-Meier curves for T stage (T4 vs. T3/2/1) and for solid vs. non-solid histology are depicted in Figure 2. Overall, a 3-year DPFS of 67% for primary and 74% for postoperative RT (*p* = 0.27) and a 3-year OS of 64% for primary and 79% for postoperative RT (*p* < 0.01) could be found. Corresponding Kaplan-Meier estimates for OS and DPFS are depicted in Figure 1b,c. For potential risk factors for both time-to-event data, please see Figure S1.

**Figure 3:**
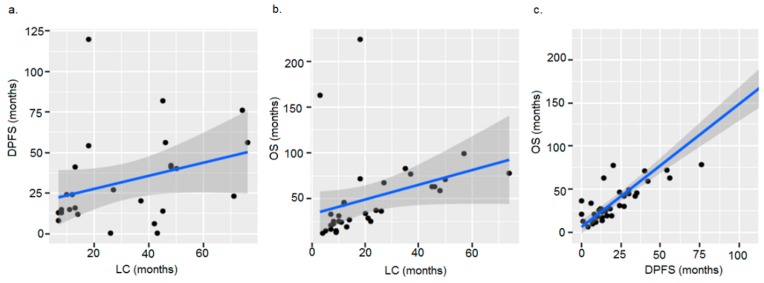
Correlation between local relapse and distant relapse (**a**), local relapse and overall survival (**b**) as well as distant relapse and overall survival (**c**) over time in months. Abbreviations: LC = local control, DPFS = distant progression-free survival, OS = overall survival.

Linear regression analysis showed, that the time to local relapse explains more of the variance observed in OS (*p* < 0.001, adjusted R^2^ = 0.759; Figure 3b) than DPFS (*p* = 0.11, adjusted R^2^ = 0.061; Figure 3a). The most significant correlation was observed between DPFS and OS (*p* < 0.001, adjusted R^2^ = 0.792; Figure 3c).

### 3.1. Failure Patterns 

Local failure occurred in a median of 28 months after the completion of RT (range 6–82 months) and distant failure in a median of 36 months after the first diagnosis (range 0–102 months). Overall, 26.9% of the patients showed a local recurrence (n = 61), 31.3% of the patients a distant failure (n = 71) and 0.8% of the patients a nodal failure (n = 2; Figure S2). The majority of local recurrences occurred in-field (n = 27/61, 44.3%) or in the area, where critical structures, i.e. skull base (n = 11/61, 18%) and orbit (n = 12/61, 19.7%), were spared (n = 25/61, 41%). Regarding distant failure, pulmonary metastases dominated (n = 28/71, 39.4%), while polytopic distant failure occurred in 43.7% of the cases (n = 32/71). For detailed analysis of the recurrence patterns, please see Table S3.

### 3.2. Toxicity Analysis

**Figure 4:**
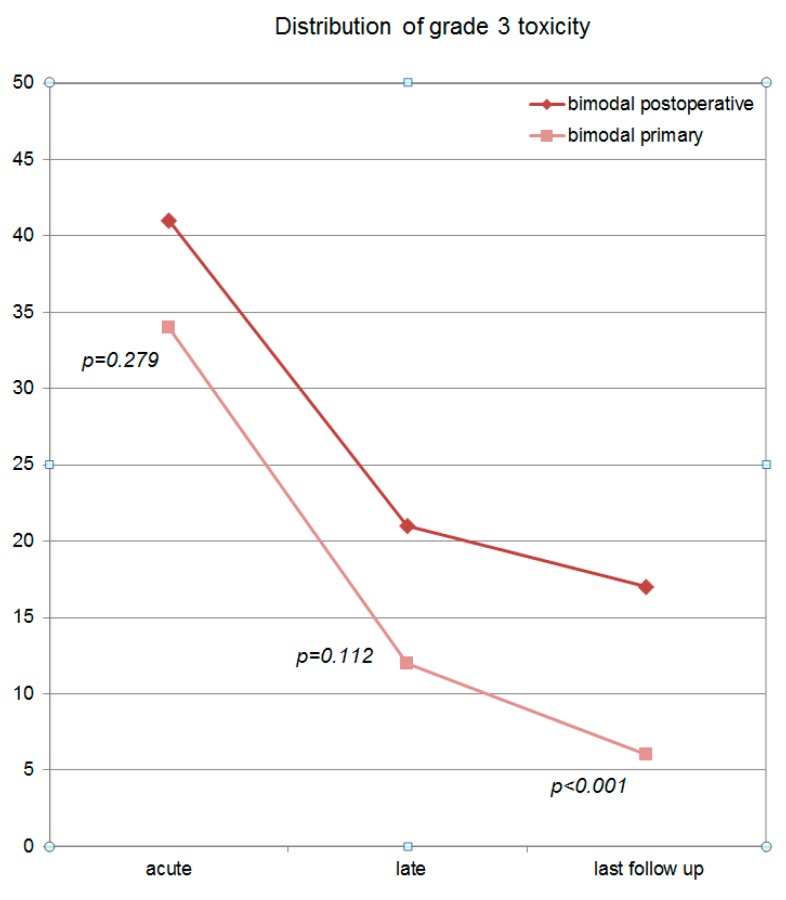
Distribution of grade 3 toxicity (acute, late and at last follow-up) for the postoperative bimodal and the primary bimodal RT group. Postoperative bimodal RT results in significant more grade 3 toxicity over time (*p* < 0.01).

Overall, 34.4% of the patients in the primary (n = 31/90) and 41.6% of the patients in the postoperative bimodal RT group (n = 57/137) claimed acute grade 3 toxicity. Predominantly, grade 3 acute mucositis, dys-/odynophagia and dysgeusia/dysosmia were reported (for details, please see Table S4). At last follow-up, the majority of these symptoms resolved. As reported, grade 3 acute toxicity was high and decreased over follow-up (Figure 4) for both treatment groups. At last follow-up, grade 3 chronic toxicity was observed in 6.2% of the patients in the primary (n = 5/81) and in 17.2% of the patients in the postoperative bimodal RT group (n = 21/122). At all three time points (acute, late, at last follow-up), more grade 3 toxicity occurred in patients, who received postoperative RT vs. definitive bimodal RT (*p = 0.279* for acute grade 3 toxicity, *p* = 0.112 for late grade 3 toxicity and *p* < 0.001 for long-term grade 3 toxicity at last follow-up; Figure 4). The major long-term grade 3 toxicities which occurred predominantly in postoperatively treated patients were xerostomia (n = 16, 13.1%), osteoradionecrosis (n = 2, 2%) and wound healing disorder (n = 2, 2%).

## 4. Discussion

### 4.1. Findings

Although paranasal ACC is a rare malignancy [33], we treated 227 patients with ACC of the paranasal sinuses within the last 10 years. The aim of the current study was to describe long-term results and furthermore to compare treatment outcome of primary and postoperative bimodal RT for these patients. The authors showed that primary RT in sinonasal ACC patients treated in a bimodal setting with IMRT and carbon ion boost results in adequate LC without any statistical differences in the LC rate of primary vs. postoperatively treated patients. This can possibly be explained by the high rate of macroscopic residual disease (R2) in the postoperative RT group (63%) [34]. Solid histology and T4 stage could be additionally identified as independent prognostic factors for decreased local control. 

Although similar LC rates could be identified for both treatment methods, postoperative RT resulted in a significantly higher OS rate compared to the primary RT group, possibly explained by a negative patient selection for primary RT (inoperable tumors, patients with comorbidity, T4 stage, larger tumor volumes (CTV2)). Several studies associated age, upper T stage, N+ stage and solid histology with the development of distant metastases and worse prognosis [35]. In the current study as well, the authors could identify a significant correlation between time of distant failure and death. While recent developments, e.g., IMRT and high-LET (linear energy transfer) radiotherapy [36], resulted in considerably increased LC rates, distant control, most frequently involving the lungs and survival still remains challenging due to missing systemic treatment options [34]. Several systemic approaches to improve the prognosis of ACC patients are investigated in the primary setting but the most active systemic treatment, which was established in the 1980s, still remains the CAP chemotherapy in selected patients [37,38,39]. Nevertheless, the high rate of local and distant relapses in ACC patients limiting survival requires the identification of new systemic treatment approaches. 

The incidence of positive neck lymph nodes in ACC patients is low and ranges between 5% and 15% in dependence of the tumor location [40,41,42]. In addition, due to the anatomical lymphatic drainage, tumors of the paranasal sinuses seem to spread less common into the regional lymph nodes including level I (especially in case of infiltration of the nasal cavity), II and III as well as the parapharyngeal neck nodes [43,44,45] compared to other sites in the head and neck region [43]. In the current study, 12% of the patients were initially diagnosed with cervical lymph node metastases (n = 28/227) and only two patients developed nodal failure during follow-up (both in-field, n = 2/28, 7%). No patient who received elective RT of the cervical lymphatic drainage for advanced tumor stages (T3/4) showed nodal failure after radiotherapy. On the basis of these excellent results, we would recommend elective RT of the unilateral cervical neck nodes in case of unilaterally seated tumors and of the bilateral cervical lymph nodes in case of midline infiltrating tumors for advanced tumor stages (T3/4). Nevertheless, considering the overall low lymph node involvement of ACCs, the elective RT of the lymphatic drainage should be reconsidered individually in order to spare toxicity.

Finally, acute and late grade 3 toxicity were assessed and compared between the two treatment groups. At each time point, postoperatively irradiated patients, as expected, showed more severe toxicity, but only long-term grade 3 toxicity at last follow-up differed significantly between the two groups.

### 4.2. Primary vs. Postoperative Radiotherapy

Especially in the area of the paranasal sinuses, surgery is extremely challenging due to the proximity of the skull base, palate and surrounding soft tissue, that the majority of patients with paranasal ACC has gross residual tumors, making LC difficult [16,19]. In a case series of 309 ACC patients with 116 paranasal ACC (37%), Jensen et al. published first long-term results of bimodal RT for these tumors (1998–2013) over a time period of fifteen years, predominantly data from the GSI, Darmstadt (1998–2009) [34]. Overall, only 28% of the patients received a primary bimodal and 73% of the patients a postoperative bimodal RT. The majority of patients had locally advanced tumors (T4a/b 60%) or was irradiated for a macroscopic tumor disease due to a high rate of subtotal resection (72%). Consequently, limited LC with a 5-year LC rate of 59% was described for these tumors. In the current study, an estimated 5-year LC of 58% for primary bimodal and 65% for postoperative bimodal RT could be achieved in paranasal ACC patients (*p = 0.33*). In the literature, no further data comparing primary and postoperative RT for high-LET radiotherapy in paranasal ACC patients are available. Several recent data regarding photon beam radiotherapy for ACC of the head and neck reported inferior LC for primary irradiated patients vs. postoperatively irradiated patients [8,15,46], which could not be shown for dose-escalated RT with carbon ion boost in the current study although a trend towards better LC can be suspected with longer follow-up. Thus, Mendenhall et al. reported a similar 5–year LC rate of 56% for RT alone vs. a much higher 5–year LC rate of 94% for postoperative RT in 101 patients with ACC of the minor and major salivary glands in the head and neck treated with photon or electron beam radiotherapy [8]. The differences in LC for postoperatively irradiated patients between Mendenhall´s and our results can possibly be explained by selection bias and especially the high rate of macroscopic residual disease after surgery in our patient collective.

### 4.3. Prognostic Factors

Surgery represents the standard treatment for ACC. Nevertheless, the majority of ACC patients receive primary RT for locally advanced tumor stages or postoperative RT, when risk factors for decreased LC, i.e., R+, PNI, LVI, N+ are known [4,7,8,47,48]. While several authors described macroscopic tumor disease as an unfavorable prognostic factor for LC [4,5,8,13,29], Jensen et al. could show in a prospective phase-II trial, that resection status for ACC of the head and neck had no impact on LC, perhaps due to the dose-escalation effect of carbon ions [24]. Only patients, who were treated for locally recurrent ACCs, could be identified with worse LC by Jensen et al. vs. patients, who received primary treatment. In several studies, T4 stage was associated with worse LC. While overall LC for photon RT ranged between 38% and 64% at five years for locally advanced tumors, LC for T4 tumors was 14% to 44% [4,5,8,9,14]. Nevertheless, for carbon ions as well, several authors described T stage as an independent prognostic factor for decreased LC with a 5-year LC of 100% in T1, 80.2% in T2, 72.5% in T3, 70.9% in T4a and 38.6% in T4b [34]. In addition to T4 stage, we identified solid histology as further independent factor for decreased LC, which was reported by several other authors as well [35].

### 4.4. Toxicity

In contrast to photon beam RT, high-LET radiotherapy with carbon ions enables dose-escalation by higher relative biological effectiveness due to the biological advantages of carbon ions. Additionally, the active-raster scanning technique, as used in the current study, guarantees the formation of extremely sharp dose gradients for very precise dose delivery [49]. Therefore, dose coverage of the target volume can be maintained, while critical organs at risk are preserved sufficiently. In the prospective COSMIC trial by Jensen et al., the authors described an acute grade 3 mucositis rate of 26%, an acute grade 3 dermatitis rate of 6%, an acute tympanic effusion in 30% of the patients as well as a chronic hearing impairment in 25%, late grade 1 and 2 brain necrosis in 6%, late grade 4 hemorrhage of the internal carotid artery in 2%, facial nerve paralysis in 2% and late osteoradionecrosis in 4% of the patients for dose-escalated bimodal treatment up to doses >80 Gy (EQD2) [24]. Mucositis and hearing problems were reported to be the most common acute grade 3 toxicities, comparable with prior results for dose-escalated bimodal as well as photon beam RT, generally applied in doses up to 66 Gy (EQD2) maximum [14,22,50]. In the current study as well, a high mucositis rate of 17.8% for primary and 17.5% for postoperative RT was observed. Concerning carotid blowout syndrome, cranial nerve paralysis, osteoradionecrosis and brain injury, several authors described a dose-dependence, while the probability for these symptoms rises strongly with higher RT doses >70 Gy [51,52,53,54,55,56]. Although high doses (median total EQD2 of 80 Gy) were applied in the current study, no grade 3 cranial nerve affection or radiogenic brain injury occurred. Osteoradionecrosis grade 3 and higher grade of visual impairment could be identified each in only two patients. Overall, toxicity of bimodal RT is comparable with other carbon ion data despite high-doses (median total EQD2 of 80 Gy) and lower than reported proton and neutron data [16,18,19,20,21,25,54,57]. Although no significant differences between primary bimodal and postoperative bimodal RT were identified for acute and late grade 3 toxicity, patients in the postoperative bimodal RT group showed significant more grade 3 long-term toxicity (toxicity at last follow-up), which consisted in the most cases of a grade 3 xerostomia (13.1%). Several studies in the last decades have still shown a high morbidity rate of combination treatment with surgery and RT for different entities and different locations. 

### 4.5. Limitations and Outlook

The major limitations of the current retrospective study are of methodological nature, as patient records may contain insufficient, incomplete or inaccurate data causing selection and information bias. Consequently, survival results and prognostic factors should be considered critically under this aspect. In addition, the lack of sufficiently powered studies on the basis of the rareness of ACCs prohibits making general treatment recommendations which are valid internationally. As an example, the extension of the planning target volume especially in terms of including or not including the elective neck area seem to be an unresolved question managed differently in each center. The variety of treatment planning concepts, treatment modalities (photon RT, proton RT, carbon ion RT) and dose calculation models for carbon ion RT (e.g., Japan vs. Germany) make a clear comparison of the treatment results described in the literature as well as the determination of the role of adding carbon ions as boost to a photon base plan difficult. Although bimodal RT including IMRT and carbon ion RT delivers promising results compared to photon beam RT alone, this treatment method is only available in Germany. Generally, the availability of carbon ion treatment centers is strongly limited to few centers worldwide [36]. Additionally, the role of alternative fractionation schedules, i.e. accelerated RT, remains unclear due to missing data.

## 5. Conclusions

Primary radiotherapy in a bimodal setting including IMRT and carbon ion boost for dose-escalation results in adequate LC with less long-term grade 3 toxicity compared to postoperative bimodal radiotherapy in sinonasal ACC patients. The high rate of macroscopic tumor disease in the postoperative group makes the interpretation of the beneficial results in LC for primary RT difficult. Therefore, randomized trials are required.

## Figures and Tables

**Table 1:** Patient, tumor and treatment characteristics.

Patient Characteristic	*No. (%)*			*p-*Value
	Overall (n = 227)	Primary Bimodal (n = 90)	Postop. Bimodal (n = 137)	Primary vs. Postoperative
**Sex**				0.102
male	118 (52.0)	40 (44.4)	78 (56.9)	
female	109 (48.0)	50 (55.5)	59 (43.1)	
**Median age at first diagnosis**	55 years	56years	53 years	0.184
range	(17–80 years)	(21–80 years)	(17–77 years)	
**Karnofsky performance status**				0.920
100	39 (17.1)	15 (16.7)	24 (17.5)	
90	116 (51.1)	47 (52.2)	69 (50.4)	
80	52 (22.9)	19 (21.1)	33 (24.1)	
≤70	20 (8.8)	9 (10.0)	11 (8.0)	
**Tumor location**				0.157
maxillary sinus	122 (53.7)	42 (46.7)	80 (58.4)	
sphenoid sinus	18 (7.9)	11 (12.2)	7 (5.1)	
ethmoid sinus	6 (2.2)	1 (1.1)	4 (2.9)	
frontal sinus	1 (0.4)	none	1 (0.7)	
infiltration of ≥2 sinuses	81 (35.7)	36 (40.0)	45 (32.8)	
**Tumor side**				0.318
unilateral	148 (65.2)	55 (61.1)	93 (67.9)	
bilateral (midline involving)	79 (34.8)	35 (38.9)	44 (32.1)	
**Histological subtype (of n = 197)**				0.624
solid	73 (32.1)	32 (35.6)	40 (29.2)	
non-solid	96 (42.3)	36 (40.0)	60 (43.8)	
mixed	43 (18.9)	16 (17.8)	27 (19.7)	
kribriform	48 (21.1)	19 (21.1)	29 (21.2)	
tubular	3 (1.3)	none	3 (2.2)	
trabecular	2 (0.8)	none	2 (1.5)	
serous	1 (0.4)	1 (1.1)	none	
unknown	58 (25.6)	22 (24.4)	36 (26.3)	
**TNM classification**				
**T stage**				0.012 *
1	2 (0.8)	2 (2.2)	none	
2	9 (4.0)	none	9 (6.6)	
3	36 (15.9)	9 (10.0)	27 (19.7)	
4	180 (79.3)	79 (87.8)	101 (73.7)	
**N stage**				0.008
0	199 (87.7)	77 (85.6)	122 (89.1)	
1	10 (4.4)	4 (4.4)	6 (4.4)	
2	18 (7.9)	9 (10.0)	9 (6.6)	
**M stage**				0.561
0	224 (98.7)	88 (97.8)	136 (99.3)	
1	3 (1.3)	2 (2.2)	1 (0.7)	
**G stage**				0.686
1	13 (5.7)	5 (5.5)	8 (5.8)	
2	34 (15.0)	9 (10.0)	25 (18.2)	
3	13 (5.7)	5 (5.6)	8 (5.8)	
X	167 (73.6)	71 (78.9)	96 (70.1)	
				
**LVPn**				0.497
0	11 (4.8)	2 (2.2)	9 (6.6)	
1	97 (42.7)	32 (35.6)	65 (47.4)	
X	119 (52.4)	56 (62.2)	63 (46.0)	
**R stage**				NA
R0	15 (6.6)	none	15 (10.9)	
R1	35 (15.4)	none	35 (25.5)	
R2	86 (37.9)	none	86 (62.8)	
**Concomitant systemic therapy with cetuximab**	8 (3.5)	4 (4.4)	4 (2.9)	NA
**Treatment volume**				
median CTV1	390 cc (100–1247 cc)	392 cc (100–1247 cc)	390 cc (100–1116 cc)	0.728
median CTV2	175 cc (28–647 cc)	181 cc (28–647 cc)	170 cc (44–396 cc)	0.196
**Treatment doses**				
EQD2 CTV1	50 Gy (48 Gy–56 Gy)	50 Gy (48 Gy–56 Gy)	50 Gy (48 Gy–56 Gy)	0.016
EQD2 CTV2	80 Gy (71 Gy– 80 Gy)	80 Gy (71 Gy–80 Gy)	80 Gy (73 Gy–80 Gy)	0.781

Abbreviations: RT = radiotherapy, postop. = postoperative, CTV1 = clinical target volume including CTV2 and the elective lymphatic drainage, CTV2 = clinical target volume including the tumor or the tumor bed, G = grading, LVPn = lymphovascular and perineural invasion, R stage = resection stage, EQD2 = equivalent dose in 2 Gy single dose fractions, * T4 vs. T3/2/1.

**Table 2:** Multivariate analysis for local control.

Variable	HR (95%-CI)	*p-*Value
primary vs. postop.	1.057 (0.574–1.947)	0.859
solid vs. non-solid histology	2.350 (1.287–4.290)	0.005
T4 vs. T3/T2/T1	10.021 (2.411–41.662)	0.002

Abbreviations: HR= hazard ratio, CI= confidence interval, KPS= Karnofsky Performance score.

## Supplementary Materials

The following are available online at https://www.mdpi.com/2072-6694/11/11/1705/s1, Figure S1: Distribution of the risk factors age., Figure S2. Venn diagram showing the distribution and the overlap of local recurrences., Table S1: RECIST analysis of 227 patients at different time points over follow-up., Table S2: Univariate analysis for local control., Table S3: Analysis of the recurrence patterns at last follow-up., Table S4: Overview of acute and late toxicity over time.
