# Supplementary material for: Treatment Outcome of 227 Patients with Sinonasal Adenoid Cystic Carcinoma (ACC) after Intensity Modulated Radiotherapy and Active Raster-Scanning Carbon Ion Boost: A 10-Year Single-Center Experience
Source: Cancers (Basel). 2019 Nov 1;11(11):1705. doi: 10.3390/cancers11111705 (PMC6895865; doi:10.3390/cancers11111705)
Supplement: Supplementary file 1 [file cancers-11-01705-s001.pdf]

*Supplementary material*

# Treatment Outcome of 227 Patients with Sinonasal Adenoid Cystic Carcinoma (ACC) after Intensity Modulated Radiotherapy and Active Raster-Scanning Carbon Ion Boost: A 10-Year Single-Center Experience

**Sati Akbaba; Dina Ahmed; Andreas Mock; Thomas Held; Suzan Bahadir; Kristin Lang; Mustafa Syed; Juliane Hoerner-Rieber; Tobias Forster; Philippe Federspil; Klaus Herfarth; Peter Plinkert; Juergen Debus; Sebastian Adeberg**

**Table S1.** RECIST analysis of 227 patients at different time points over follow-up.

| Follow-Up Time | No. (%) | | | | |
|---|---|---|---|---|---|
| | **3 Months Post RT** | **6 Months Post RT** | **12 Months Post RT** | **24 Months Post RT** | **At Last Follow-Up** |
| **Data excluded** | **19/227 (8.4)** | **25/227 (11.0)** | **39/227 (17.2)** | **67/227 (29.5)** | **55/227 (24.2)** |
| Loss to follow-up | 17/227 (7.5) | 17/227(7.5) | 20/227 (8.8) | 30/227 (13.2) | 32/227 (14.1) |
| Deaths without local recurrence | 1/227 (0.4) | 5/227 (2.2) | 11/227 (4.8) | 19/227 (8.4) | 23/227 (10.1) |
| Follow-up time has not been reached | 1/227 (0.4) | 3/227 (1.3) | 8/227 (3.5) | 18/227 (7.9) | none |
| **Data available** | **208/227 (91.6)** | **202/227 (89.0)** | **188/227 (82.8)** | **160/227 (70.5)** | **172/227 (75.8)** |
| SD | 48/208 (23.1) | 34/202 (16.8) | 25/188 (13.3) | 17/160 (10.6) | 9/172 (5.2) |
| PR | 76/208 (35.5) | 78/202 (38.6) | 63/188 (33.5) | 42/160 (26.3) | 25/172 (14.5) |
| CR | 76/208 (35.5) | 78/202 (38.6) | 80/188 (42.6) | 70/160 (43.8) | 55/172 (32.0) |
| Recurrence | 8/208 (3.8) | 14/202 (6.9) | 20/188 (10.6) | 31/160 (19.4) | 61/172 (35.5) |

Abbreviations: RECIST= Response Evaluation Criteria in Solid Tumors, RT= radiotherapy, SD= stable disease, PR= partial remission, CR= complete remission

**Table S2. Univariate analysis for local control**

| **Variable** | **HR (95%-CI)** | *p*-value |
|---|---|---|
| primary vs. postop. | 1.297 (0.768-12.193) | 0.330 |
| age (10 year steps) | 0.959 (0.792-1.160) | 0.664 |
| KPS ≤ 70 vs. KPS >70 | 2.105 (0.946-4.681) | **0.068** |
| solid vs. non-solid histology | 2.143 (1.181-3.887) | **0.010** |
| T4 vs. T3/T2/T1 | 6.786 (2.121-21.71) | **<0.001** |
| midline involvement vs. unilateral | 1.186 (0.691-2.035) | 0.536 |
| G3 vs. G2/G1 | 2.115 (0.746-5.991) | 0.149 |
| LVPn1 vs. LVPn0 | * | * |

**Table S2.** *Cont.*

| | | |
|---|---|---|
| R1 vs. R0 | 4.450 (0.563-35.16) | 0.157 |
| R2 vs. R0 | 5.183 (0.703-38.22) | 0.106 |

Abbreviations: HR= hazard ratio, CI= confidence interval, KPS= Karnofsky Performance score
* Could not be reliably calculated due to lack of events

**Table S3: Analysis of the recurrence patterns at last follow-up**

| Pattern of Local Recurrence | No. of Patients (n=61, %) |
|---|---|
| in-field | 27 (44.3%) |
| out-of-field | 4 (6.6%) |
| pterygopalatine fossa* | 1 (1.6) |
| cavernous sinus* | 2 (3.3) |
| infratemporal fossa** | 1 (1.6) |
| at critical structures | 25 (41.0) |
| skull base | 11 (18.0) |
| brain stem | 1 (1.6) |
| cavernous sinus | 7 (11.5) |
| temporal lobe | 1 (1.6) |
| clivus | 2 (3.3) |
| orbit | 12 (19.7) |
| inner ear | 1 (1.6) |
| sinus ethmoidalis | 1 (1.6) |
| no information available | 5 (8.2) |
| **Pattern of distant recurrence** | **No. of patients (n=71, %)** |
| cerebral | 5 (7.0) |
| pulmonary | 28 (39.4) |
| osseous | 4 (5.6) |
| brain stem | 2 (2.8) |
| polytopic | 32 (43.7) |
| **Pattern of nodal recurrence** | **No. of patients (n=2, %)** |
| in-field | 2 (100) |
| out-of-field (contralateral) | none |

Abbreviations: RT= radiotherapy, *tumor spread via the maxillary nerve, ** tumor spread via the mandibular nerve

**Table S4:** Overview of acute and late toxicity over time

| | **Acute** | | **Chronic** | | **At Last Follow-Up** | |
|---|---|---|---|---|---|---|
| | **definitive RT (n=90)** | **postop. RT (n=137)** | **definitive RT (n=81)** | **postop. RT (n=122)** | **definitive RT (n=81)** | **postop. RT (n=122)** |
| overall toxicity | | | | | | |
| grade ≤2 | 59 (65.5) | 80 (58.4) | 71 (87.7) | 96 (78.7) | 76 (93.8) | 101 (82.8) |
| grade 3 | 31 (34.4) | 57 (41.6) | 10 (12.3) | 26 (21.3) | 5 (6.2) | 21 (17.2) |
| mucositis | | | | | | |
| grade ≤2 | 56 (62.2) | 85 (62.0) | 1 (1.2) | 4 (32.8) | none | none |
| grade 3 | 16 (17.8) | 24 (17.5) | none | none | none | none |
| dermatitis | | | | | | |
| grade ≤2 | 66 (73.3) | 98 (71.3) | 14 (17.3) | 22 (18.0) | none | 4 (3.3) |
| grade 3 | 1 (1.1) | 1 (0.7) | none | none | none | none |
| oral thrush | | | | | | |
| grade ≤2 | 12 (13.3) | 12 (9.5) | 2 (2.5) | none | none | none |
| grade 3 | 1 (1.1) | none | none | none | none | none |
| dysgeusia/dysosmia | | | | | | |
| grade ≤2 | 54 (60.0) | 81 (59.1) | 34 (42.0) | 60 (49.2) | 8 (9.9) | 27 (22.1) |
| dys-/odynophagia | | | | | | |
| grade ≤2 | 45 (50.0) | 58 (42.3) | 7 (8.6) | 10 (8.2) | 1 (1.2) | 5 (4.1) |
| grade 3 | 8 (8.9) | 10 (7.3) | 1 (1.2) | 1 (0.8) | none | 1 (0.8) |
| xersotomia | | | | | | |
| grade ≤2 | 44 (48.9) | 57 (41.6) | 23 (28.4) | 42 (34.4) | 6 (7.4) | 15 (12.3) |
| grade 3 | 13 (14.4) | 35 (25.5) | 7 (8.6) | 24 (19.7) | 4 (4.9) | 16 (13.1) |
| gastric tube dependence | 6 (6.7) | 11 (8.0) | 1 (1.2) | 3 (2.5) | none | 1 (0.8) |
| trismus | | | | | | |
| grade ≤2 | 13 (14.4) | 29 (21.2) | 14 (17.3) | 29 (23.8) | 1 (1.2) | 17 (13.9) |
| grade 3 | none | 1 (0.7) | none | 2 (1.6) | none | 1 (0.8) |
| keratokonjunktivitis sicca | | | | | | |
| grade ≤2 | 6 (6.7) | 41 (29.9) | 17 (21.0) | 19 (15.6) | 2 (2.5) | 6 (4.9) |
| grade 3 | none | 1 (0.7) | none | none | none | none |
| rhinitis (sicca) | | | | | | |
| grade ≤2 | 7 (7.8) | 21 (15.3) | 9 (11.1) | 27 (22.1) | 2 (2.5) | 9 (7.4) |
| radiogenic brain necrosis | | | | | | |
| grade ≤2 | none | none | 2 (2.5) | 2 (1.6) | 5 (6.2) | 6 (4.9) |
| osteoradionecrosis | | | | | | |

| | | | | | | |
|---|---|---|---|---|---|---|
| grade ≤2 | none | none | 3 (3.7) | 4 (3.3) | 3 (3.7) | 4 (3.3) |
| grade 3 | none | none | none | 1 (0.8) | none | 2 (1.6) |
| visual impairment | | | | | | |
| grade ≤2 | 5 (5.6) | 10 (7.3) | 7 (8.6) | 8 (6.6) | 4 (4.9) | 1 (0.8) |
| grade 3 | none | none | 1 (1.2) | none | none | 1 (0.8) |

**Table S4.** Cont.

| | | | | | | |
|---|---|---|---|---|---|---|
| conductive hearing impairment | | | | | | |
| grade ≤2 | 14 (15.5) | 13 (9.5) | 5 (6.2) | 11 (9.0) | none | 2 (1.6) |
| grade 3 | 1 (1.1) | 1 (0.7) | 2 (2.5) | none | none | 1 (0.8) |
| tympanic effusion | | | | | | |
| grade ≤2 | 9 (10.0) | 26 (19.0) | 8 (9.9) | 13 (10.7) | 4 (4.9) | 3 (2.5) |
| neuropathic pain | | | | | | |
| grade ≤2 | 2 (2.2) | 2 (1.5) | 2 (2.5) | 1 (0.8) | none | 1 (0.8) |
| cranial nerve affection | | | | | | |
| optical nerve grad 1 | none | none | none | 1 (0.8) | none | 1 (0.8) |
| trigeminal nerve grade1 | 3 (3.3) | 1 (0.7) | 1 (1.2) | none | none | 1 (0.8) |
| facial nerve grade 1 | 2 (2.2) | 8 (5.8) | 9 (11.1) | 7 (5.7) | 2 (2.5) | 3 (2.5) |
| vestibulocochlear nerve | 2 (2.2) | 2 (1.5) | 4 (4.9) | 3 (2.5) | 4 (4.9) | 3 (2.5) |
| hearing loss grade ≤2 | 1 (1.1) | 2 (1.5) | 1 (1.2) | 3 (2.5) | 1 (1.2) | 2 (1.6) |
| hearing loss grade 3 | none | none | 2 (2.5) | none | 2 (2.5) | none |
| vertigo grade ≤2 | 2 (2.2) | none | 4 (4.9) | 2 (1.6) | 4 (4.9) | 2 (1.6) |
| hypoglossal nerve grade 1 | none | none | 1 (1.2) | none | 1 (1.2) | none |
| recurrent laryngeal nerve grade1 | none | none | 1 (1.2) | none | 1 (1.2) | none |
| wound complications | | | | | | |
| grade 3 | 1 (1.1) | 1 (0.7) | none | 2 (1.6) | none | 2 (1.6) |

Abbreviations: RT= radiotherapy, postop.= postoperative

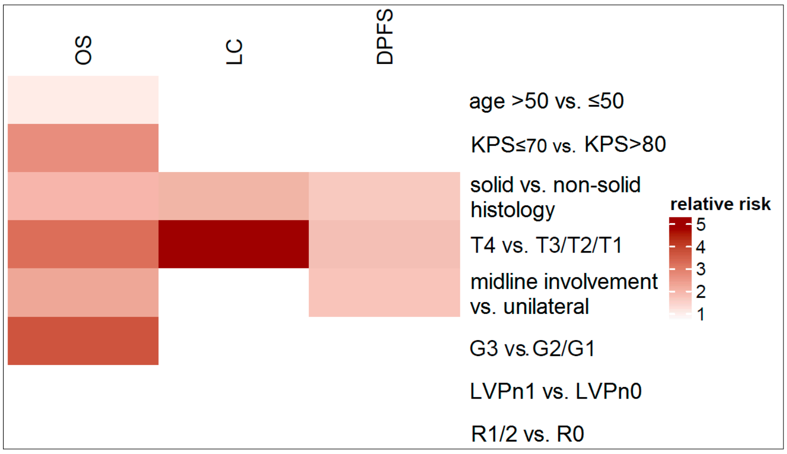


**Figure S1.** Distribution of the risk factors age, Karnofsky performance score (KPS), solid vs. non-solid histology, T stage, unilateral vs. midline involvement, G stage, LVPn stage and R stage, for overall survival (OS), local control (LC) and distant progression-free survival (DPFS). The individual risk factors are weighted with different colors depending on their intensity of influence (relative risk of 1 to 5).*abbreviations: OS = overall survival, LC = local control, DPFS = distant progression-free survival, KPS = Karnofsky Performance Status, LVPn = lymphovascular and perineural invasion, R = resection margin

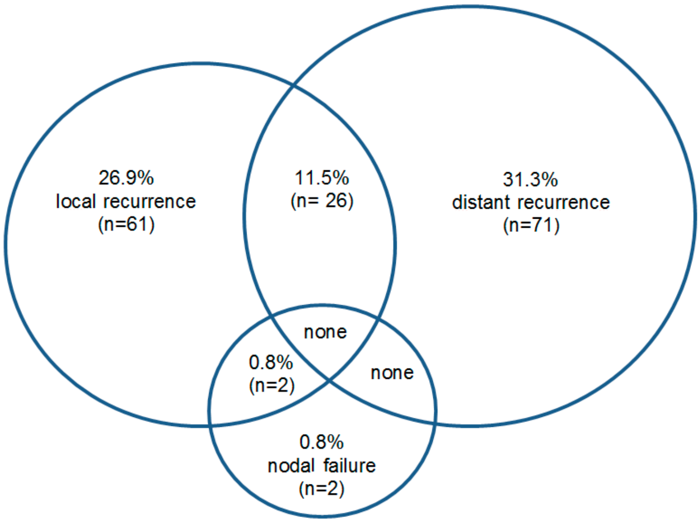


**Figure S2.** Venn diagram showing the distribution and the overlap of local recurrences, distant recurrences and nodal failure for the whole patient collective after bimodal radiotherapy for paranasal adenoid cystic carcinoma patients.

© 2019 by the authors. Licensee MDPI, Basel, Switzerland. This article is an open access article distributed under the terms and conditions of the Creative Commons Attribution (CC BY) license (http://creativecommons.org/licenses/by/4.0/).
